# Machine-Learning Classification of a Number of Contaminant Sources in an Urban Water Network

**DOI:** 10.3390/s21010245

**Published:** 2021-01-01

**Authors:** Ivana Lučin, Luka Grbčić, Zoran Čarija, Lado Kranjčević

**Affiliations:** 1 Faculty of Engineering, University of Rijeka, Vukovarska 58, 51000 Rijeka, Croatia; lgrbcic@riteh.hr (L.G.); zcarija@riteh.hr (Z.Č.); lado.kranjcevic@riteh.hr (L.K.); 2 Center for Advanced Computing and Modelling, University of Rijeka, Radmile Matejčić 2, 51000 Rijeka, Croatia

**Keywords:** water distribution networks, water network contamination, machine learning, random forest, neural network

## Abstract

In the case of a contamination event in water distribution networks, several studies have considered different methods to determine contamination scenario information. It would be greatly beneficial to know the exact number of contaminant injection locations since some methods can only be applied in the case of a single injection location and others have greater efficiency. In this work, the Neural Network and Random Forest classifying algorithms are used to predict the number of contaminant injection locations. The prediction model is trained with data obtained from simulated contamination event scenarios with random injection starting time, duration, concentration value, and the number of injection locations which varies from 1 to 4. Classification is made to determine if single or multiple injection locations occurred, and to predict the exact number of injection locations. Data was obtained for two different benchmark networks, medium-sized network Net3 and large-sized Richmond network. Additionally, an investigation of sensor layouts, demand uncertainty, and fuzzy sensors on model accuracy is conducted. The proposed approach shows excellent accuracy in predicting if single or multiple contaminant injections in a water supply network occurred and good accuracy for the exact number of injection locations.

## 1. Introduction

Contamination in water distribution networks can occur due to deliberate or unintentional intrusions and it is of extreme importance to determine the contamination event parameters so it can be detected which parts of water distribution networks have been exposed to the contaminant and needed measures can be conducted. This is considered to be an inverse problem since injection location, injection starting time, injection duration, and contaminant chemical concentration value needs to be predicted based on sensor measurements. Numerical simulations are used to determine these parameters, but model limitations need to be taken into consideration. EPANET [1] is the most commonly used software for water distribution network simulations and uses an advective approach which cannot efficiently analyze contaminant dispersion in the networks. Piazza et al. [2] conducted experiments where it was shown that dispersive and diffusive processes must be incorporated in the transport model for less turbulent fluid flows to achieve more accurate results than the pure advection model. Also, EPANET assumes complete mixing in all network junctions, which can be valid only in the case of a single outlet or if there is considerable distance between two junctions. Therefore, EPANET extension EPANET-BAM [3] was proposed which uses experimentally calibrated mixing model parameter to more accurately model mixing in network junctions. A number of studies investigated mixing behavior for different conditions, both experimentally and numerically, to further enhance these simpler 1D numerical models [4,5,6,7,8,9].

Huang and McBean [10] investigated a data mining approach for identifying possible sources of intrusion where single and multiple injection scenarios were considered. In the case of multiple injection scenario, the method provided a limited number of nodes with the probability of them being the true contamination source. However, in their work, it is not predicted what is the true number of injection locations. In Wang and Harrison [11] a Bayesian approach was coupled with Support Vector Regression to provide a probability distribution of water network nodes being contaminant sources. However, a single injection is assumed, and it is noted that multiple contaminant sources should be considered in future work where the likelihood evaluation needs to be adjusted. Seth et al. [12] investigated the efficiency of three different methods for source detection; Bayesian probability-based method, backtracking method (using contaminant status algorithm), and optimization-based method where accuracy in case of multiple injection locations was investigated for two and three contamination injection locations. It was noted that the Bayesian method is designed only for a single contamination location while the contaminant status algorithm used in De Sanctis et al. [13] provides a list of possible solutions that narrow down search space for the optimization method; however, it also does not identify the possible number of injection locations. In Lučin et al. [14] a new search space reduction method was proposed, which can eliminate a considerable number of source nodes for both single and multiple injection locations, but with considerably greater reduction for single injection scenario. A number of different optimization approaches were considered to determine the contamination source, an overview of proposed methods can be found in Adedoja et al. [15]. Optimization approach can be easily extended to consider multiple contamination sources, as mentioned in [16,17,18].

If considering the optimization approach with multiple injection locations, with each additional source of contamination, the complexity of search space increases with an increase of optimization variables. Since the number of injection locations is not known, as a precaution, multiple injection locations should be allowed, since optimization can set variables to zero (which eliminates that source node and eliminates the number of injection locations), but it cannot add additional variables (injection locations) during the optimization process. In this way, in the case of a single injection location, optimization can eliminate other source nodes (all contamination parameters would be set to 0). However, this considerably increases the complexity of the considered problem since unnecessary fitness function evaluations would be conducted due to greater search space. Thus, it would be greatly beneficial to determine the number of injection locations before the optimization algorithm is employed. Also, if it is known that a single injection event occurred, a number of methods can be used more efficiently to reduce the complexity of the problem. For example, the machine learning approach provides probabilities for each network node being the true contamination scenario, which greatly reduces the number of suspect nodes and helps in quicker detection of true contamination location. However, in the case of multiple injections, different likelihood evaluation is needed which increases the complexity of the machine learning approach. Prediction of the number of contamination sources has previously been conducted for air pollution in Wade and Senocak [19], but to authors knowledge was not conducted for water distribution network contamination scenarios.

Machine learning tools have been increasingly used in contamination detection, where Random Forest has been used for groundwater source of contamination detection [20] and source detection in a river [21]. In Grbčić et al. [22] Random Forest algorithm was used to predict contamination event parameters in water distribution networks and in Grbčić et al. [23] new machine learning-based algorithm was proposed. A great advantage of prediction models is that they can be constructed before an accident occurs, so when a contamination event is detected prediction can be made even for large networks in a computationally efficient way. Thus, the proposed model which predicts number of injection locations can be used prior to conducting approaches that search for contamination parameters, without influencing the reaction time needed to contain the contamination event. However, in accident situations hydraulic conditions can greatly differ from those on which model was trained, thus, a wrong prediction could be made. This can be handled with the preparation of multiple prediction models with different hydraulic conditions or by using a prediction model that achieves great accuracy with the small number of inputs so time for prediction also becomes negligible considering the benefit of search space reduction when redundant optimization parameters are not used.

In this paper, the Random Forest and Artificial Neural Network classifier are used to predict the number of contamination sources based on contamination sensor measurements in the water distribution network. Sensor measurements of contamination needed for model teaching are obtained from contamination scenarios simulated using EPANET2 with Monte Carlo generated contamination parameters. An investigation was conducted for two different sized benchmark water distribution networks with different sensor layouts, to examine the efficiency of the proposed machine learning approach. Investigation of demand uncertainty and fuzzy sensors is also estimated.

## 2. Materials and Methods

### 2.1. Benchmark Water Supply Networks

**Figure 1:**
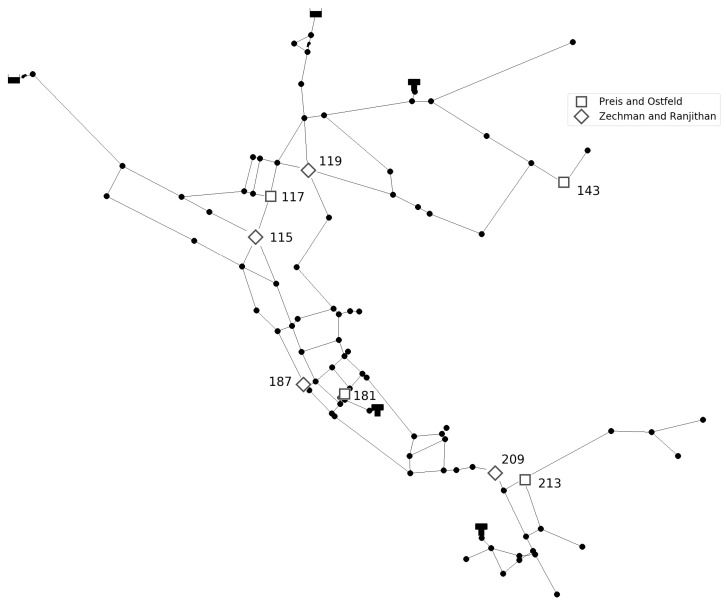
Net3 network with sensor layouts.

**Figure 2:**
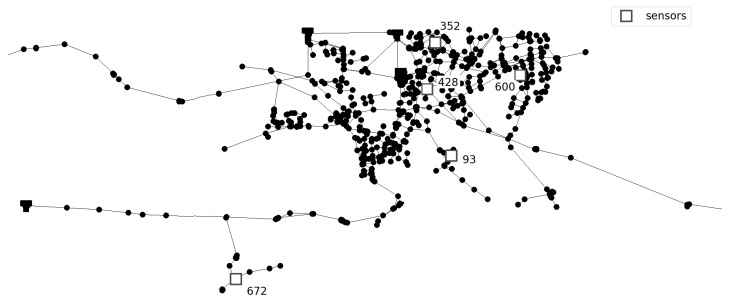
Richmond network detail with sensor layout.

Prediction of the number of injection sources is conducted for two benchmark different sized networks. Investigated networks are Net3 EPANET2 example consisting of 92 nodes and Richmond network consisting of 865 nodes, obtained from The Centre for Water Systems (CWS) at the University of Exeter [24]. For the Net3 network, two different sensor layouts are investigated. In first layout four sensors were placed in network nodes 117, 143, 181, and 213 as in [25] and in second layout four sensor were placed in network nodes 115, 119, 187, and 209 as in [26]. Additionally, an investigation of the number of sensors was conducted. For the first layout, two sensors were placed in network nodes 117 and 181, and for the second layout sensors were placed in network nodes 119 and 209. For Richmond network five sensors were placed in network nodes 93, 352, 428, 600, and 672 where sensor layout was taken from [27]. Layout with three sensors placed in network nodes 93, 428, and 672 was also considered. Considered networks with sensor layouts can be seen in Figure 1 and Figure 2.

Contamination scenarios are simulated using EPANET2 version 2.0.12. where for both networks, simulation time is 24 h with a hydraulic time step of 10 min, quality time step 5 min, pattern time step 10 min and report time step 1 h. For all conducted simulations, the EPANET2 flow paced method is used for the contaminant injection. Contamination scenario parameters are chosen randomly. The number of injection locations is chosen from 1 to 4 nodes. The starting time and duration of contamination injection are chosen from 0 to 24 h. Concentration was randomly chosen from 10 to 2000 mg/L. For contamination scenarios with multiple injection locations starting time, duration, and concentration was kept the same for every injection location.

Prior to simulating multiple injection scenario, independent simulations for each randomly chosen node as a source of contamination are conducted. If contamination is not registered for the investigated node with chosen contamination parameters, that node is eliminated as source location and only nodes for which contamination was detected in at least one sensor are kept as a source of contaminant. For example, if four source nodes are randomly chosen to be the source of contamination, but only two source nodes influence sensor detection of contaminant, the same time series of sensor measurements would be obtained for two, three, and four injection locations since the latter two do not influence contamination measurements. If four sources are given to the prediction model as input, where contamination can be measured only from two sources, that would significantly reduce the accuracy of the prediction model. Thus, only nodes which contribute to the contamination measurements in sensors are considered for multiple injection scenario.

**Figure 3:**
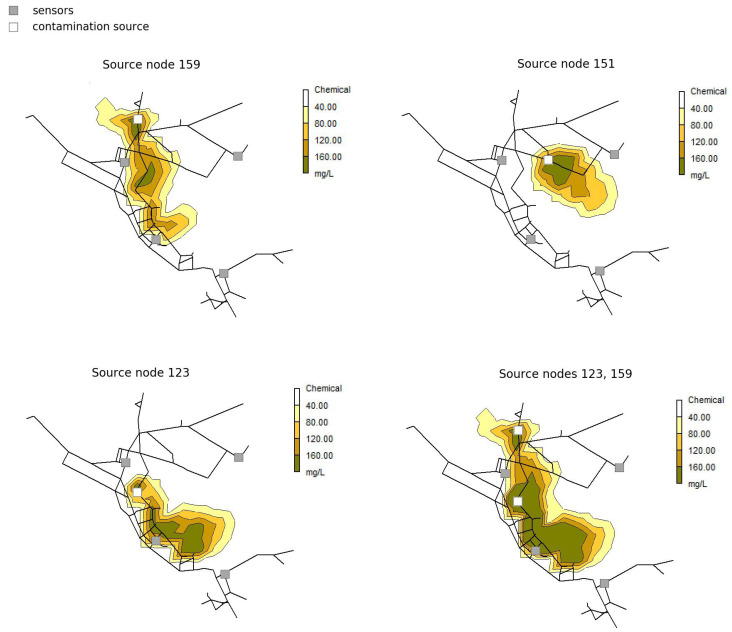
Contours of chemical for randomly chosen Net3 contamination scenario 90 min after injection starting time. Contamination from source node 151 remains undetected, so the source node is not included for multiple injections scenario.

**Figure 4:**
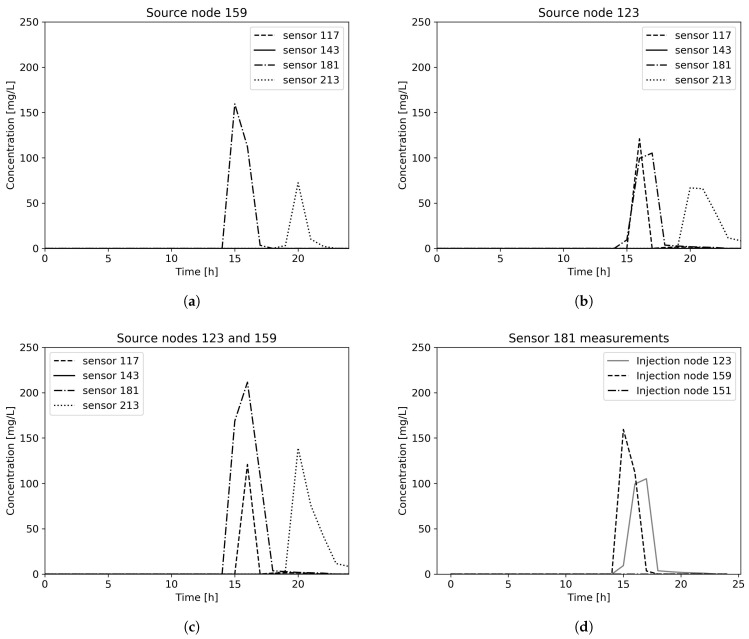
Sensor measurements for Net3 contamination scenario with (**a**) injection node 159, (**b**) injection node 123, (**c**) injection nodes 123 and 159 and (**d**) contamination measurements in the sensor in node 181.

An example of the proposed methodology can be seen for arbitrarily chosen Net3 contamination scenario in Figure 3. Randomly chosen contamination scenario parameters are 3 source nodes (159, 151 and 123), with contamination value of 200 mg/L, starting time 13 h and 20 min and injection duration 2 h. Sensor measurements for chosen contamination scenario can be seen in Figure 4. It can be observed that for source node 151 contamination scenario remains undetected in all sensors placed in the water distribution network, thus for multiple sources scenario only source nodes 123 and 159 are further considered.

### 2.2. Demand Uncertainty and Sensor Type

To investigate demand uncertainty, for both Net3 and Richmond networks, for every network node first it was randomly chosen if demand will be altered or not. If node base demand was to be altered, the percentage from 0–5% is randomly chosen for each network node, to reduce or increase base demand by the chosen percentage, resulting in a random demand span of 10%. To further investigate influence of demand uncertainty, the percentage from 0–10% is randomly chosen to reduce or increase base demand, resulting in a random demand span of 20%. All network demand patterns were kept the same, only base demand was changed. This method was conducted for every contamination scenario, thus resulting in different hydraulic conditions for each contamination scenario.

For sensor type influence, fuzzy sensor measurements were made where sensor detection was considered either low, medium, or high. Chemical concentration value *C* in range 0<C<300 mg/L was considered low, in range 300<C<1000 mg/L was considered medium and high if C>1000 mg/L. Prediction model input features were defined as 0 if no contaminant was detected, 1 for low measurements, 2 and 3 for medium and high measurements, respectively.

### 2.3. Machine Learning Classifiers

**Figure 5:**
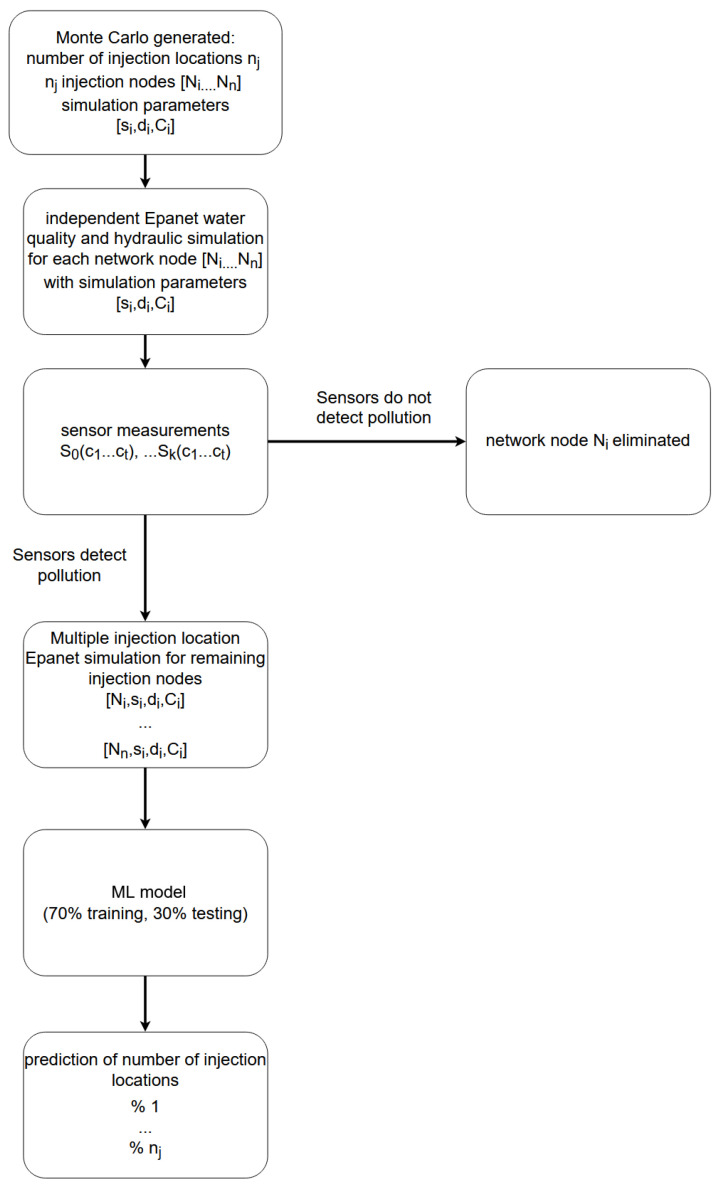
Flowchart of Machine Learning algorithm for prediction of number of contamination sources.

Two different machine learning classifiers, Random Forest and Artificial Neural Network were used to compare the efficiency of the proposed method. Random Forest algorithm [28], based on multiple decision trees is used, with 250 estimators (trees) with a maximum depth of 30 and the minimum number of samples required to split an internal node 8. An artificial neural network with three hidden layers with 100 nodes in each layer, with hyperbolic tangent activation function and Adam solver for weight optimization is used. Proposed parameters were chosen with the grid search hyperparameter optimization method, while other parameters, which are not mentioned, are kept constant. Implementation in the Python library Scikit-learn [29] version 0.20.3 is used for both classifiers. Obtained data was split 70% for teaching and 30% for model testing. Flowchart of the prediction model can be seen in Figure 5. Data generation and prediction model training was done using the supercomputing resources at the Center for Advanced Computing and Modelling, University of Rijeka.

Input data for the prediction model is the time series of sensor measurements. For both Net3 and Richmond network, 25 features per sensor are obtained, which resulted in 100 features for Net3 and 125 features for Richmond network. The output of the machine learning model is the number of injection locations where two different prediction models are used. The first prediction model was used to predict the exact number of injection locations, i.e., 4 different classes are predicted. In the second model it is predicted only if single or multiple injections occurred, i.e., 2, 3 and 4 injection locations are treated as same, multiple injections class, thus only 2 different classes are predicted (single and multiple injections). To further increase the accuracy of the latter prediction model, the threshold value is introduced. Only if the model predicts a single source scenario with a probability greater than the chosen threshold value, single source prediction is made. In other cases, the scenario is treated as multiple sources. Threshold values of 50%, 60%, 70%, 80%, 90%, and 95% are investigated.

## 3. Results

### 3.1. Model Accuracy

**Figure 6:**
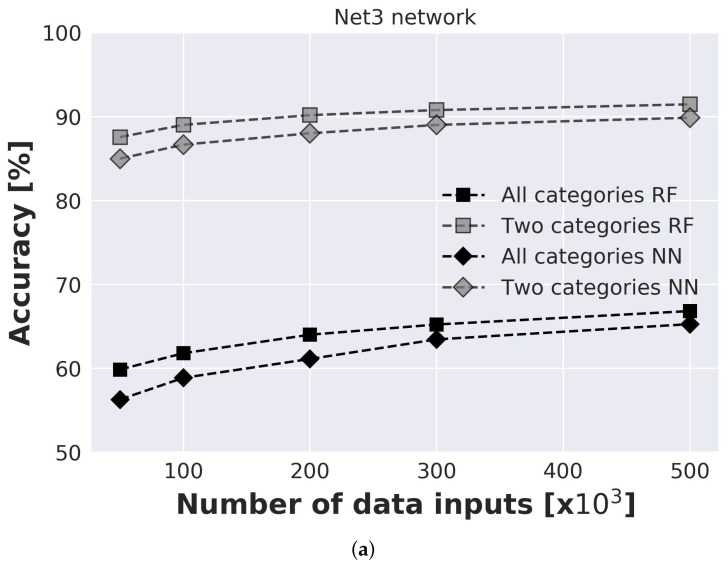

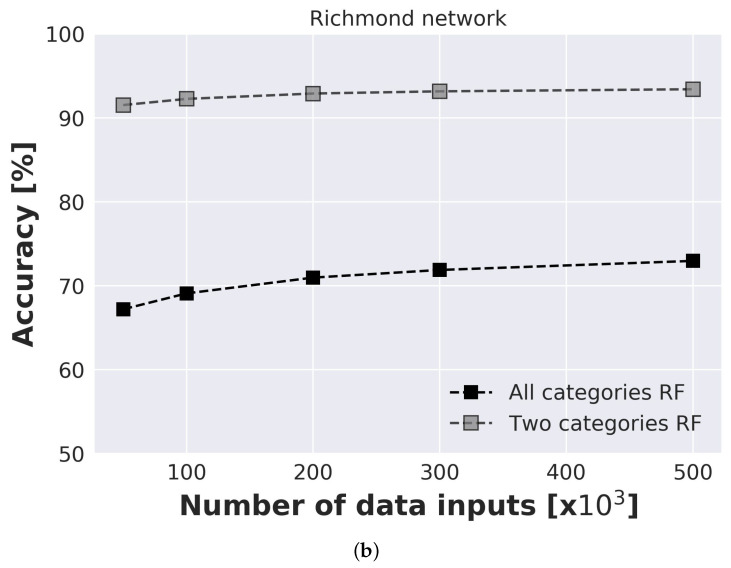
Accuracy of prediction models for different number of inputs for (**a**) Net3 network and (**b**) Richmond network.

The influence of input data on prediction model accuracy is investigated for both benchmark networks where data ranged from 50,000 to 500,000 inputs (Figure 6). An investigation is conducted for prediction model with 2 categories (model predicts only if single or multiple injection locations are present) and with 4 categories (model predicts an exact number of injection locations). For each model and each number of inputs, 20 runs were conducted to take into consideration the influence of random seed. For the Net3 network second sensor layout with sensors placed in nodes 115, 119, 187, and 209 was considered. For Net 3 results are presented for both RF and NN prediction models. Standard deviation ranged from 0.63% for 50,000 to 0.33% for 500,000 inputs for NN model, and from 0.33% for 50,000 to 0.1% for 500,000 inputs. It can be observed that the RF model has slightly better accuracy for all investigated models. Also, due to the faster execution time of the RF model, for all further analyses, only RF results will be presented. For Richmond network, standard deviation ranged from 0.28% for 50,000 inputs to 0.12% for 500,000 inputs which indicates the stability of the model. Presented results are an average of all 20 runs.

It can be observed that even for a small number of input data considerable accuracy can be achieved. For model with 2 categories even with 50,000 inputs accuracy of the model is above 85% for both considered networks. After 200,000 inputs accuracy of the models for both networks tend to only slightly increase with the further increase of the number of input data. For 500,000 inputs accuracy of the Net3 network is 66.83% and for Richmond network 72.96%. When simplification is made, and the model only needs to predict single or multiple injection locations, accuracy significantly increases and for 500,000 inputs for the Net3 network is 91.46% and for the Richmond network 93.4%.

### 3.2. Threshold Influence

To further increase the accuracy of the prediction model, the threshold value is introduced for the model which predicts 2 categories. Detailed results are presented for models with 500,000 inputs for Net3 (Table 1 and Table 2) and Richmond network (Table 3 and Table 4). Presented results are the average of values obtained from 20 runs. As expected, with the increase in threshold value accuracy of the prediction model increases. However, with a greater threshold value, a greater number of single injection scenarios, as a precaution, are classified as multiple sources, thus a smaller number of true single injection scenarios are detected. For both networks, when the threshold value is 95%, a very low percentage of correct prediction of single source scenarios can be observed when prediction model parameters chosen with grid search optimization method (250 estimators, maximum depth 30, minimum samples for split 8) were used (Table 1 and Table 3). Thus, different prediction model parameters (180 estimators, maximum depth 80, minimum samples for split 10) were also investigated to test its influence on model accuracy when threshold values are considered. In Table 2 and Table 4 it can be observed that for the greatest threshold value (95%) correct prediction of single sources scenarios greatly increases, and is around 30% of the total number of single source scenarios. As threshold value decreases, similar percentages are observed for both models, which indicates that model accuracy is similar for different RF parameters. However, when greater prediction certainty is expected, model parameters must be carefully considered.

For both networks, accuracy with threshold value 95% is above 99.5%. It can be observed from Table 2 that for Net3 only 36% of total number of single source scenarios are correctly predicted where for Richmond network (Table 4) that value is 37%. For threshold value 50% for Net3 94.5% of single injection scenarios are correctly predicted; however, the number of wrong predictions increases. The same can be observed for the Richmond network where for threshold value 50%, 97.8% of single injection scenarios are correctly predicted but the percentage of wrong single injection scenarios increases from 0.8% to 12.7%.

The problem remains with scenarios that are wrongly predicted even for a threshold value of 95%. With further increase of threshold value, the number of wrongly predicted scenarios would decrease, but only because ultimately all scenarios would be classified as multiple sources (this can also be observed in Table 1 and Table 3 for first chosen RF parameters). Thus, optimum threshold value should be chosen to both provide a reasonable number of single injection scenario predictions but with a high model accuracy. In-depth analysis of scenarios where the model wrongly predicts a single injection scenario with a high threshold value should be conducted. Also, it should be investigated how much accuracy of the model can be further increased with a larger number of inputs and with the usage of different classifiers.

### 3.3. Sensor Layout

The influence of sensor layout was tested for both Net3 and Richmond networks. 20 runs were conducted for the model with 500,000 inputs and average accuracy for all runs can be seen in Table 5. It can be observed that for the same number of sensors, their layout influences the accuracy of prediction models. This is expected, since the same behavior can be seen when the detection rate of contamination event is investigated for different sensor layouts. In the paper by Ostfeld et al. [30] for the same network and the same number of sensors detection likelihood of contamination event greatly differs for different sensor layouts. Results show that the prediction model for 2 categories (predicts single or multiple injections) is less influenced by sensor layout and all sensor layouts have accuracy around 90% or higher.

Interestingly, greater model accuracy can be observed when a smaller number of sensors is placed for Net3 layout with sensors in nodes 117, 143, 181, and 213 and for Richmond network. However, it can be explained with the fact that a greater number of contamination events remain undetected. I.e., with the greater number of sensors, contamination events from the greater number of network nodes are detected, resulting in more combinations when considering multiple injection locations. When sensor placement is sparser, a smaller number of network nodes can be detected when the contamination event occurs, resulting in a smaller number of combinations for multiple injection locations and consequently providing better model accuracy with 500,000 inputs.

### 3.4. Demand Uncertainty and Fuzzy Sensors

Influence of demand uncertainty and fuzzy sensors was investigated for Net3 network with 4 sensors in nodes 117, 143, 181 and 213 and for Richmond network with 5 sensors in nodes 93, 352, 428, 600 and 672. 20 runs were conducted for RF models with 500,000 inputs and average accuracy can be observed in Table 6. When demand uncertainty is considered the accuracy of RF models slightly decreases for both networks. The influence of fuzzy sensors is more prominent, where the greater reduction in prediction accuracy can be observed for the Net3 network. When considering both demand uncertainty and fuzzy sensors in the same model, accuracy further slightly decreases. However, it can be observed that for both networks model which predicts 2 categories has accuracy above 90% for all cases. This shows that the proposed model could be applied in a real case scenario.

## 4. Discussion

Accuracy of prediction models for both networks has similar results with small differences, which shows that the proposed methodology could be successfully applied to other networks. Further investigation should be conducted for large size water distribution networks and different sensor placements, to fully investigate the robustness of the proposed method. Also, it must be noted that simplification was used in this study, where all source nodes had the same parameters (injection starting time, duration, and concentration value), thus, it should be investigated how the model predicts if those parameters are different for each injection node.

Although slightly, with the increase of input data model accuracy still increases, so in further study a greater number of data inputs should be investigated. Also, in the proposed scenarios report time step was chosen to be 1 h, resulting in 25 features per sensor. It should be investigated if a greater number of features, i.e., smaller report time step would increase model accuracy and if similar model accuracy could be achieved with a smaller number of contamination readings. The optimal number of features and inputs should be investigated to achieve great accuracy but with reasonable execution time. However, to obtain a greater number of inputs a greater amount of time is needed, so the model should be trained before the actual contamination event occurs. In that case, the model would be trained with simulation results with average demand patterns. This surely would mean that true contamination event will have different demands which would influence the accuracy of the prediction model. Investigation of demand uncertainty with arbitrarily chosen demand variation spans showed that small differences of base demands slightly influence prediction model accuracy. However, it must be taken into consideration that when base demand variation is defined with percentage, small demand variation is achieved when base demand is small and greater demand variation only when base demand is greater. Greater difference in demands should be further investigated since the usual variability of consumption can be greater than considered in this paper. Different machine learning models, with different expected demand patterns, can be prepared for contamination event so prediction can be obtained instantaneously. However, in case of contamination event, greater oscillations in the hydraulics of water distribution network could occur, such as pipe burst or some other unplanned event, which would greatly influence change in demand patterns. Thus, it would be beneficial to investigate other algorithms that could increase accuracy with a smaller number of input data. In that case, input data can be obtained after the contamination event occurred, in a reasonable amount of time. That would be greatly beneficial since the simulation model can then be calibrated with sensor measurements from the field and input data would be more precise. The proposed method can be easily coupled with other machine learning approaches since inputs obtained for this model can also be used for teaching model that predicts injection location.

Investigation of different sensor layouts, demand uncertainty, and fuzzy sensors showed that sensor layout and type of sensors have the greatest impact on prediction model accuracy. Demand uncertainty slightly decreases model accuracy. However, model accuracy can be greatly reduced when a real case event is considered since both demand uncertainty and measurement errors can be greater than considered in this work. Thus, a threshold value is introduced which can help increase model accuracy. Greater threshold value increases model accuracy; however, it also leads to a greater number of single injection scenarios classified as multiple injections. It is also observed that prediction models are not very sensitive to model parameters; however, when threshold value is used, i.e., model prediction certainty is evaluated, model parameters are very important for method efficiency. Thus, the investigation of different machine learning approaches should be further investigated to increase model accuracy.

When observing presented results it must be taken into consideration that numerical model simplifications are made, where EPANET was used which assumes complete mixing in all network junctions and uses pure advection transport model. Also, in the presented study benchmark networks are used, and numerical simulations are conducted for only 24 h, where more than 24 h are needed to obtain stable contamination scenario results. However, the functionality of the presented machine learning approach is not dependent on the numerical model setup, and it is assumed that the same numerical approach that is chosen for the optimization process is to be also chosen for the prediction model preparation. In this way, all discrepancies due to numerical model simplifications would be also present in the optimization and as such are not the result of using the proposed machine learning approach. Furthermore, network uncertainties were not considered regarding internal pipe diameter and pipe roughness which should be considered in the further research.

## 5. Conclusions

In this paper, the machine learning approach is presented which helps identify the number of injection locations based on sensor measurements. Random Forest classifier and Neural Network classifier are used on medium-sized benchmark network, where Random Forest classifier provided better accuracy and faster execution time, thus is used for all other investigations. Two different sized benchmark networks are considered, where it is shown that the machine learning approach can be successfully used to predict the number of injection locations. This can help define the number of optimization parameters, where redundant parameters can be avoided which needlessly increase the complexity of the problem. The prediction model shows great accuracy when it predicts only if single or multiple injection locations occurred. The threshold value is proposed which further increases model accuracy since the single injection scenario is assumed only if the model predicts with certainty greater than the threshold value. Lower accuracy is obtained when the exact number of injection locations is predicted. The accuracy of the prediction model is investigated for different sensor layouts and in case of demand uncertainties and fuzzy sensors. Conducted research showed promising results, where exploration of other algorithms and increased number of input data should be investigated to further increase the accuracy of both models.

## Figures and Tables

**Table 1:** Influence of threshold value on model accuracy for Net3 network (250 estimators, maximum depth 30, minimum samples for split 8). Percentage indicates number of predicted simulations based on total number of single source scenarios.

Threshold Value	Accuracy	Single Source Scenarios	Correct Prediction	Wrong Prediction
95%	99.98%	48,682	3307 (6.8%)	36 (0.07%)
90%	99.73%	48,682	15,388 (31.6%)	405 (0.8%)
80%	98.6%	48,682	34,717 (71.3%)	2085 (4.3%)
70%	97.5%	48,682	41,204 (84.6%)	3683 (7.6%)
60%	96.7%	48,682	44,334 (91.1%)	4914 (10.1%)
50%	95.7%	48,682	46,388 (95.3%)	6390 (13.1%)

**Table 2:** Influence of threshold value on model accuracy for Net3 network (180 estimators, maximum depth 80, minimum samples for split 10). Percentage indicates number of predicted simulations based on total number of single source scenarios.

Threshold Value	Accuracy	Single Source Scenarios	Correct Prediction	Wrong Prediction
95%	99.7%	48,783	17,458 (35.8%)	508 (1%)
90%	99.4%	48,783	25,426 (52.1%)	863 (1.8%)
80%	98.9%	48,783	35,197 (72.2%)	1667 (3.4%)
70%	98.2%	48,783	40,640 (83.3%)	2636 (5.4%)
60%	96.7%	48,783	43,977 (90.2%)	3737 (7.7%)
50%	95.7%	48,783	46,091 (94.5%)	5072 (10.4%)

**Table 3:** Influence of threshold value on model accuracy for Richmond network (250 estimators, maximum depth 30, minimum samples for split 8). Percentage indicates number of predicted simulations based on total number of single source scenarios.

Threshold Value	Accuracy	Single Source Scenarios	Correct Prediction	Wrong Prediction
95%	99.9%	52,911	375 (0.7%)	5 (0.001%)
90%	99.8%	52,911	10,889 (20.6%)	303 (0.6%)
80%	97.9%	52,911	37,463 (70.8%)	3076 (5.8%)
70%	95.8%	52,911	49,149 (92.9%)	6269 (11.9%)
60%	94.8%	52,911	51,427 (97.2%)	7819 (14.8%)
50%	93.9%	52,911	52,198 (98.65%)	9178 (17.3%)

**Table 4:** Influence of threshold value on model accuracy for Richmond network (180 estimators, maximum depth 80, minimum samples for split 10). Percentage indicates number of predicted simulations based on total number of single source scenarios.

Threshold Value	Accuracy	Single Source Scenarios	Correct Prediction	Wrong Prediction
95%	99.7%	52,941	19,499 (36.8%)	435 (0.8%)
90%	99.3%	52,941	30,305 (57.2%)	1085 (2.1%)
80%	98.3%	52,941	42,000 (79.3%)	2567 (4.9%)
70%	97.3%	52,941	47,654 (90%)	4061 (7.7%)
60%	96.4%	52,941	50,433 (95.3%)	5433 (10.3%)
50%	95.5%	52,941	51,775 (97.8%)	6703 (12.7%)

**Table 5:** Influence of sensor layout for Net3 and Richmond networks on prediction model accuracy.

	Sensors Locations	Accuracy	
		4 Categories	2 Categories
Net3	117, 143, 181, 213	71%	94%
	115, 119, 187, 209	67%	91%
	117, 181	75%	89%
	119, 209	63%	89%
Richmond	93, 352, 428, 600, 672	73%	93%
	93, 428, 672	83%	92%

**Table 6:** Influence of demand uncertainty and fuzzy sensors for Net3 and Richmond network on prediction model accuracy.

	**Net3**	
	**4 Categories**	**2 Categories**
perfect sensors	71%	94%
demand uncertainty (±5%)	69%	93%
demand uncertainty (±10%)	69%	93%
fuzzy sensors	65%	91%
demand uncertainty (±5%) and fuzzy sensors	64%	90%
demand uncertainty (±10%) and fuzzy sensors	63%	90%
	**Richmond**	
	**4 Categories**	**2 Categories**
perfect sensors	73%	93%
demand uncertainty (±5%)	72%	93%
demand uncertainty (±10%)	72%	93%
fuzzy sensors	72%	93%
demand uncertainty (±5%) and fuzzy sensors	71%	93%
demand uncertainty (±10%) and fuzzy sensors	71%	92%

## Data Availability

The data presented in this study are available on request from the corresponding author.
